# Sensitive Detection of *Plasmodium vivax* Using a High-Throughput, Colourimetric Loop Mediated Isothermal Amplification (HtLAMP) Platform: A Potential Novel Tool for Malaria Elimination

**DOI:** 10.1371/journal.pntd.0004443

**Published:** 2016-02-12

**Authors:** Sumudu Britton, Qin Cheng, Matthew J. Grigg, Catherine B. Poole, Cielo Pasay, Timothy William, Kimberley Fornace, Nicholas M. Anstey, Colin J. Sutherland, Chris Drakeley, James S. McCarthy

**Affiliations:** 1 University of Queensland, Brisbane, Australia and QIMR Berghofer Medical Research Institute, Brisbane, Australia; 2 Australian Army Malaria Institute, Brisbane, Australia; 3 Menzies School of Health Research and Charles Darwin University, Darwin, Australia; 4 New England Biolabs, Ipswich, Massachusetts, United States of America; 5 Jesselton Medical Centre, Kota Kinabalu, Sabah, Malaysia; 6 London School of Hygiene and Tropical Medicine, London, United Kingdom; Walter and Eliza Hall Institute, AUSTRALIA

## Abstract

**Introduction:**

*Plasmodium vivax* malaria has a wide geographic distribution and poses challenges to malaria elimination that are likely to be greater than those of *P*. *falciparum*. Diagnostic tools for *P*. *vivax* infection in non-reference laboratory settings are limited to microscopy and rapid diagnostic tests but these are unreliable at low parasitemia. The development and validation of a high-throughput and sensitive assay for *P*. *vivax* is a priority.

**Methods:**

A high-throughput LAMP assay targeting a *P*. *vivax* mitochondrial gene and deploying colorimetric detection in a 96-well plate format was developed and evaluated in the laboratory. Diagnostic accuracy was compared against microscopy, antigen detection tests and PCR and validated in samples from malaria patients and community controls in a district hospital setting in Sabah, Malaysia.

**Results:**

The high throughput LAMP-*P*. *vivax* assay (HtLAMP-Pv) performed with an estimated limit of detection of 1.4 parasites/ μL. Assay primers demonstrated cross-reactivity with *P*. *knowlesi* but not with other *Plasmodium* spp. Field testing of HtLAMP-Pv was conducted using 149 samples from symptomatic malaria patients (64 *P*. *vivax*, 17 *P*. *falciparum*, 56 *P*. *knowlesi*, 7 *P*. *malariae*, 1 mixed *P*. *knowlesi*/*P*. *vivax*, with 4 excluded). When compared against multiplex PCR, HtLAMP-Pv demonstrated a sensitivity for *P*. *vivax* of 95% (95% CI 87–99%); 61/64), and specificity of 100% (95% CI 86–100%); 25/25) when *P*. *knowlesi* samples were excluded. HtLAMP-Pv testing of 112 samples from asymptomatic community controls, 7 of which had submicroscopic *P*. *vivax* infections by PCR, showed a sensitivity of 71% (95% CI 29–96%; 5/7) and specificity of 93% (95% CI87-97%; 98/105).

**Conclusion:**

This novel HtLAMP-*P*. *vivax* assay has the potential to be a useful field applicable molecular diagnostic test for *P*. *vivax* infection in elimination settings.

## Author Summary

*Plasmodium vivax* has a worldwide distribution and is the second most common causative agent of human malaria. The dormant liver stage of *P*. *vivax* allows the infection to recur unless diagnosed and treated appropriately, which poses a significant challenge to the goals of malaria elimination and eradication as outlined by the WHO. Although highly sensitive molecular diagnostic tools are available in reference laboratory settings, the currently available diagnostic tools outside referral settings for the detection of *P*. *vivax* are limited to microscopy and rapid diagnostic tests, which are insufficiently sensitive for the detection of low level parasitemia particularly in asymptomatic individuals. Based on a DNA amplification technology called loop-mediated isothermal amplification (LAMP), this study describes the development and validation of a colourimetric, high throughput assay (HtLAMP) suitable for the detection of *P*. *vivax* infection in non-referral settings. The sensitivity of the assay combined with its field applicability offers the potential for it to play an important role as a diagnostic tool for the purpose of malaria elimination.

## Introduction

*Plasmodium vivax* is the most geographically widespread of the Plasmodium species that infect humans [1] and can cause severe and fatal disease [2]. In the 2014 World Malaria Report it was estimated that there were 15.8 million cases of *P*. *vivax* in 2013, accounting for 47% of malaria cases outside the African region [3]. Asymptomatic sub-microscopic *P*. *vivax* infection is commonly reported in endemic countries [4–6], accounting for on average 69.5% of *P*. *vivax* infection relative to those with patent parasitaemias from community surveys [7], compared with 50.8% for *P*. *falciparum* [8]. The parasite reservoir of *P*. *vivax* is also aided by the dormant liver stage which can cause relapsing infection, with fast gametocyte production allowing transmission earlier in the course of the disease, and the development of multidrug resistance [9] posing difficulties for both clinical management and malaria elimination goals.

In non-referral settings in elimination areas, the diagnostic tools currently available for detection of *P*. *vivax* infections for case management and surveillance are microscopy and immunochromatographic lateral-flow antigen detection in the form of “rapid diagnostic tests” (RDTs). Reference laboratories may also offer expert microscopy and PCR. The reliability of RDTs for diagnosing *P*. *vivax* infections, particularly at low level parasitemia, remains less than that for *P*. *falciparum* [4,10]. While the most recent WHO RDT testing report found the highest performing parasite lactate-dehyrogenase (pLDH) based *P*. *vivax* RDTs were equivalent to HRP2-based *P*. *falciparum* RDTs at parasitemias of 200/μL [11], they remain inadequately sensitive for the detection of lower level *P*. *vivax* and *P*. *falciparum* parasitemia in sub-patent asymptomatic individuals. While microscopy and RDT provide adequate diagnostic accuracy for case management of symptomatic patients in clinical settings, they have been shown to be inadequate in detecting a large proportion of low density *P*.*falciparum* infections in active community surveillance [12], and for mass screening and treatment programs [13]. Studies using RDTs specifically for detection of *P*. *vivax* in this context are yet to be performed, although similar results could be expected given the inherently lower parasitemias associated with *P*. *vivax* infection. Although molecular based assays such as conventional PCR are capable of detecting very low density infections, they are not suitable for large scale community surveillance due to complex procedures that do not allow provision of test results on the day of sample, expensive reagents and the requirement for specialised equipment.

Loop mediated isothermal amplification (LAMP) is a molecular diagnostic technology that has the potential to be a readily-applicable tool in settings such as malaria elimination. LAMP is an isothermal process that relies on the *Bacillus stearothermophilus* (Bst) enzyme and does not require cyclical temperature changes [14]. As such, unlike PCR, it offers an opportunity for field adaptation because of its low technology requirement. The output of a LAMP reaction can be visualised as a magnesium pyrophosphate precipitate detectable by turbidimetry [14], metal ion detectors such as calcein [15], hydroxynaphthol blue [16] and pico-green [17]. In addition to melting curve analysis [18], LAMP end products have also been visualised using a bioluminescent output in real time(BART) [19], a lateral flow dipstick [20], or a portable device with a fluorescence detecting unit (realAmp) [21]. LAMP has also recently been performed on non-instrumented nucleic acid amplification (NINA) platforms [22] improving its potential for field application.

LAMP has been shown to detect all *Plasmodium* species [23] including *P*. *knowlesi* [24,25], to be amenable to use with crudely extracted DNA from whole blood [26], and to have a limit of detection of 5 parasites/ μL for identifying *Plasmodium* genus and *P*. *falciparum* [27]. Commercially available Loopamp MALARIA detection kits (Eiken chemical co) using *Plasmodium* genus and *P*. *falciparum* have been found to perform well in regional health facilities, but were not capable of specifically detecting *P*. *vivax* [28,29]. Furthermore, for specific detection of *P*. *vivax*, the three LAMP assays published to date have had low analytic sensitivity, with reported detection limits of 100 plasmid copies/ μL for 18s rRNA target [23] (which was equivalent to 500 parasites/ μL when tested by Patel et.al [30]), 125 parasites/μL for Pvr64 target (highly conserved repeat region in *P*. *vivax* genome) [30] and 100 copies/ μL for alpha-tubulin target [31].

Here we describe the development and validation of a novel *P*. *vivax* specific LAMP assay targeting mitochondrial DNA in a high-throughput, colourimetric platform. Its performance was compared to PCR and microscopy in a district hospital, non reference laboratory setting.

## Methods

The clinical samples used retrospectively in this study to validate the *P*. *vivax* specific primers were obtained with ethics approval granted by the Malaysian Medical Research Ethics Committee, Menzies School of Health Research, Australia, and London School of Hygiene and Tropical Medicine, UK.

### Parasite samples used for validating primers and limit of detection (LOD)

Primers were tested against parasite DNA from well characterised parasite lines and clinical samples of *P*. *falciparum* (3D7), *P*. *vivax*, *P*. *malariae*, *P*. *ovale* spp. and *P*. *knowlesi* for cross-reactivity and LOD.

In order to determine the analytical sensitivity of the HtLAMP-Pv, a two-fold dilution series of a *P*. *vivax* DNA sample beginning at a starting parasitemia of 90,000 parasites/ μL (as determined by quantitative PCR) was evaluated in duplicate. HtLAMP-Pv performance was compared with that of previously published *P*. *vivax* primers [23,30] in the HtLAMP platform. In addition, a sample of starting parasitemia of 2000 parasites/ μL (as determined by expert microscopy) was serially diluted in 50% haematocrit uninfected blood. DNA from each dilution was extracted using Qiagen blood kit and tested in duplicate using the HtLAMP-Pv assay.

### Comparison between HtLAMP-Pv and RDT

The limit of detection of the HtLAMP-Pv assay was compared with the SD Bioline Pf/Pan RDT (Alere Standard Diagnostics). This RDT detects *P*. *falciparum* histidine-rich protein II (HRP-II) and *Plasmodium* lactate dehydrogenase (pLDH) with a reported sensitivity of 100% at 200 parasites/μL [11], microscopy and quantitative PCR on a blood sample obtained from a *P*. *vivax* blood stage clinical trial (ACTRN12614000930684) participant. Informed consent was obtained as per the approval of the QIMR Berghofer HREC. Briefly, a wild type *P*. *vivax* bank was produced using blood collected from a patient, who had returned to Australia from a malaria endemic country with PCR-proven *P*. *vivax* malaria infection, prior to treatment with artemether-lumefantrine. The clinical trial was performed as described [32] and a 2 ml EDTA-blood sample was collected from the clinical trial participant at peak parasitemia prior to commencement of antimalarial treatment. This sample was serially diluted in 50% haematocrit blood and each dilution was subjected to an LDH ELISA assay, thick film blood smear for expert microscopy, an SD Bioline Pf/Pan RDT (Alere Standard Diagnostics) and 4 x 5 μL filter paper (whatman) blood spots. The filter paper blood spots and the remaining whole blood, which had been stored at -20°C, was extracted in 10 μL and 50 μL volumes using modified chelex-saponin based DNA extraction protocols as described below. The extracted DNA was stored at -20°C until performance of the HtLAMP-Pv assay.

### Diagnostic accuracy and field testing of HtLAMP-Pv

The sensitivity and specificity of the *P*. *vivax* HtLAMP was tested retrospectively on clinical samples from patients enrolled in a randomised controlled trial and case-control study performed in Sabah, Malaysia commencing in December 2012 as outlined by Grigg *et al*. [33]. Briefly, samples were collected from microscopy positive, symptomatic patients presenting as outpatients to Kota Marudu District Hospital and asymptomatic, microscopy negative, community controls as a result of reactive active case detection from within the village of a case patient from Kota Marudu district, Sabah. These were stored as 20 μL filter paper (Whatman) blood spots. A subset of 149 microscopy positive samples and 112 microscopy negative samples were used to compare the performance of the *P*. *vivax* HtLAMP (HtLAMP-Pv), with microscopy and PCR. DNA extraction of the filter paper samples from symptomatic patients and HtLAMP-Pv were performed in the Kota Marudu district hospital laboratory, with no standing molecular diagnostic capability, using a plastic bucket adapted into a water bath, a centrifuge and a portable spectrophotometer. Two local staff members were trained in the process of performing and interpreting the assay as part of its evaluation.

### DNA extraction

The *P*.*vivax* DNA used for the analysis of sensitivity was extracted from whole blood samples as per Qiagen manufacturing protocol (QIAamp DNA mini kit) with some modifications. Briefly, 500 μL of packed red cell blood sample was mixed with 500 μL of PBS. To an aliquot of 500 μL of this mix, 400 μL of Qiagen AL Lysis buffer and 40 μL of Qiagen proteinase K were added. After incubation at 56°C for 10 minutes, 400 μL of 100% ethanol was added, mixed then loaded into a spin column for centrifugation at 8,000 rpm for 1 min. The spin column was then washed once with 650 μL of AW1 and then AW2 spinning each wash at 8,000 rpm for 1 min followed by a dry spin after the AW2 wash at 13,000 rpm for 1 minute. Nucleic acid extract were eluted in 100 μL of elution buffer and stored at -20°C until LAMP reactions were performed.

*P*. *vivax DNA* from the Kota Marudu clinical filter spot samples from symptomatic patients was extracted using an established chelex protocol [34] with incubations shortened to improve turnaround time. Briefly, 6 mm filter paper punch samples were incubated in PBS containing 0.5% saponin for 2 hours at 37°C, before being centrifuged, washed in PBS, heated at 98°C for 30 minutes in 150 μL 6% chelex and centrifuged at 4,000 rpm for 3 minutes. The resultant 100 μL DNA supernatant was then stored at -20°C until analysis by PCR and LAMP.

Red cell pellet samples from asymptomatic individuals were extracted using a different chelex protocol. Briefly, 1 ml of non-ionised water was incubated with 10 μL of whole blood for 15–30 minutes at room temperature, followed by centrifugation at 10,000–15,000 x g for 3 minutes. After discarding supernatant, 200 μL of 5% chelex was added, vortexed for 5–10 seconds, incubated at 55°C for 30–90 minutes and vortexed again for 5–10 seconds. The sample was then heated for 10 minutes at 100°C, vortexed for 5–10 seconds and centrifuged for 3 minutes at 10,000–15,000 x g. The DNA supernatant was placed in a sterile microfuge tube for storage at -20°C.

Whole blood samples, for the comparative study with RDTs, were extracted using a chelex-based DNA extraction methodology [35] modified by the addition of saponin. Briefly, 10 μL of whole blood was mixed with either 200 μL of 0.5% saponin and incubated at 37°C for 30 minutes. Samples were then centrifuged, supernatant discarded and pellet heated at 98°C in 150 μL of 6% chelex for 30 minutes. The resultant supernatant was stored at -20°C. The process was also performed on 50 μL of whole blood.

### Positive control plasmid

In order to establish specificity of the *P*. *vivax* LAMP primers, a plasmid containing *P*. *vivax cox1* gene was constructed. The target region of the gene was amplified using COX1 specific PCR primers. Reactions were performed in 20 μL total volume containing 1X NH4 buffer, 2 mM MgCl2, 200 μM dNTPs, 200 μM primer mix and 0.5 U Taq polymerase (Bioline). The ~500 bp PCR product was visualised following agarose gel electrophoresis. The PCR product was purified using a commercial kit (Roche) and TA cloned using pGEM-T easy as per manufacturer instructions. Recombinant *E*. *coli* were identified by blue-white colour selection. Presence of the *P*. *vivax cox1* gene within the plasmid was confirmed by PCR and Sanger sequencing.

### Estimation of the number of mitochondrial *cox1* genes in the *P*. *vivax* genome

Copy number of the *pvcox1 gene* in the *P*. *vivax* genome was estimated by quantitative real time PCR SYBR green PCR assay using the Light Cycler 96 (Roche). Two single copy genes coding for *P*. *vivax mdr1* (GenBank Acc No. AY618622.1) and *P*. *vivax aldolase* (GenBank Acc No. AF247063) were used as reference genes to estimate the *pvcox1* copy number. PCR reactions were set up in triplicates using the Roche Fast Start Essential DNA Green Master Mix (Cat. No. 06 402 712 001), 10 μM of each primer (Table 1) and 1 μl of each DNA (cox1 plasmid, mdr/aldolase plasmid, Pv gDNA) in a 12 μl reaction volume. Cycling conditions were: 95^0^ C for 3 mins; 45 cycles of 95^0^ C 30 secs and 60^0^ C for 1 min. This was followed by melt curve analysis to confirm correct products were synthesised.

**Table 1:** Primers used to amplify Pv mdr, aldolase and cox 1 (*Source [36]).

Gene	Primer name	Sequence
pv mdr*	Pvmdr F	5’ CTGATACAAGTGAGG AAG AACTAC G 3’
	Pvmdr R	5’ GTCCACCTGACAACTTAGATGC 3’
pv aldolase*	Pvaldo F	5’ GACAGTGCCACCATCCTTACC 3’
	PvaldoR	5’ CCTTCTCAACATTCTCCTTCTTTCC 3’
***pv cox 1***	V1V2F3	5’ GGTACTGGATGGACTTTATAT 3’
	V1V2B3	5’ GGTAATGTTAATAATAGCATTACAG 3’

To assess PCR amplification efficiencies, standard curves containing five ten-fold dilutions of two plasmids containing either *pvcox* 1, or *pvmdr1* and *pvaldolase* (1:1) Suwanarusk, 2007 [36] were prepared, starting from the same initial concentration of 7.2 x10^-4^ ng/μl. Differences in CT value between *pvcox1* and *pvmdr1*/*pvaldolase* at each plasmid concentration was calculated and averaged to derive ΔCTcal. After confirming similar amplification efficiencies, genomic DNA extracted from 5 well characterised *P*. *vivax* isolates with varying DNA concentrations were used in the copy number assay. These samples were from patients enrolled in a clinical trial and surveillance study, with ethics approval granted by the Malaysian Medical Research Ethics Committee and Menzies School of Health Research, Australia, conducted in Kota Marudu district, Sabah, Malaysia[37,38]. Mean Ct values were calculated from triplicate and analysed using Graph Pad Prism (version 6). The *pvcox1* copy number in each sample was calculated as N = 2 ^ΔΔ Ct +/- SD^ i.e. N = 2 ^(CTpvmdr1-CTpvcox1)-(CTpvmdr1cal-CTpvcox1cal)^ as reported in Suwanarusk, 2007 [36].

### PCR

Nested PCR for *P*. *vivax* was performed as previously published [39]. Reactions were performed in 20 μL total volume containing 1X buffer, 2 mM MgCl2, 200 μM dNTPs, 200 μM primer mix (rPLU5new/rPLU6 for nest 1 and rVIV1/rVIV2 for nest 2) and 0.5 U Taq polymerase (Bioline). PCR products were visualised on a 2% agarose gel.

Multiplex PCR [40] for the detection of *P*. *falciparum*, *P*. *vivax*, *P*. *malariae* and *P*. *ovale* was performed on clinical samples from the 149 symptomatic case samples, with *P*. *knowlesi* confirmed using PCR as described by Imwong *et al*. [41]. Nested PCR, as described by Singh *et al*. [42], was performed on the 112 community control samples. Quantitative PCR on the *P*. *vivax* blood stage clinical trial sample was performed as previously described [32].

### High-throughput LAMP

**Fig 1:**
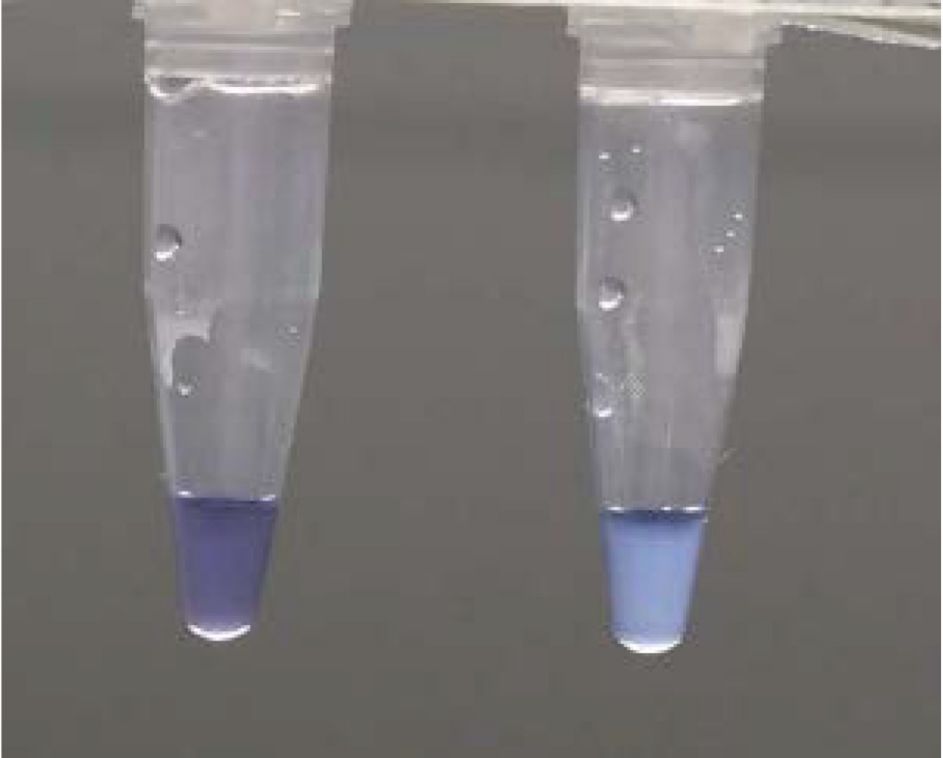
HtLAMP colour change associated with hydroxynaphtholblue (HNB). *Left* clear, purple colour = negative and *right* cloudy, blue colour = positive.

High throughput (HtLAMP) was performed on a 96-well standard u-bottom microtitre plate (Sterihealth) as previously described [43]. Briefly, reactions were performed in 25 μL total volume containing 5 μL DNA, 1X buffer (20 mM Tris HCL pH 8.8, 10 mM KCl, 8 mM MgSO_4_, 10 mM(NH_4_)SO_4_), 1.25 mM each dNTP, 1.78 μM each FIP/ BIP, 0.8 μM each LF/ LB, 0.2 μM each F3/ B3), 120 μM hydroxynaphthol-blue (Fluka, CAS number 63451-35-4)and 8 units *Bst polymerase* (New England Biolabs, Ipswich, MA). The microtitre plate was incubated in a waterbath at 65°C for 30 minutes before the colour change and precipitate in each well was recorded. A blue colour change with a visible precipitate was scored as a positive result, and purple colour without a precipitate was a negative result (Fig 1). The microtitre plate was then read in an ELISA plate reader at 600 nm wavelength to obtain an optical density (OD) reading of each well. The threshold value for a positive reaction was calculated using the mean plus two standard deviations of the no template control (NTC) wells. A positive or negative OD reading for each sample was then calculated using the threshold value and correlated with the visually detected colour change. Samples that were discordant in terms of colour change and OD threshold were deemed negative.

### Statistical analysis

PCR, nested or multiplex, for the detection of *P*. *vivax* was used as the gold standard by which the sensitivity and specificity of HtLAMP-Pv was calculated. PCR is the best established molecular diagnostic tool available for the detection of *Plasmodium* parasites and therefore an appropriate choice for comparison of a new molecular diagnostic modality.

Sensitivity was estimated as the number of LAMP positives that were also PCR positive, divided by the number of PCR positives. Specificity was estimated as the number of LAMP negatives that were also PCR negative divided by the total number of PCR negatives.

## Results

### *P*.*vivax* LAMP primers

**Fig 2:**
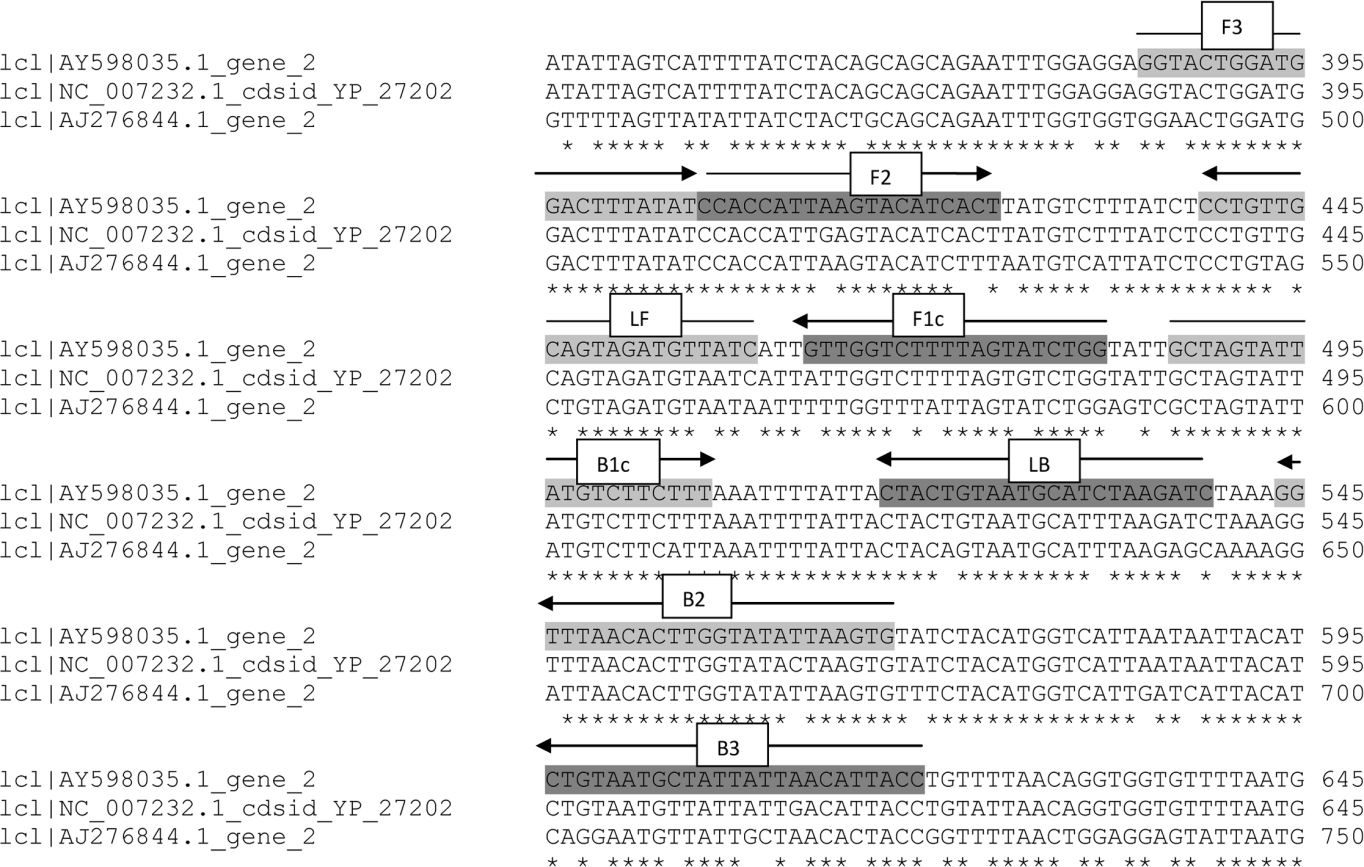
*P*. *vivax* VIV2 HtLAMP primer set superimposed on alignments of *P*. *vivax* (AY598035), *P*. *falciparum* (AJ276844) and *P*. *knowlesi* (NC_007232) *cox1* genes.

Two sets of LAMP primers (VIV1 and VIV2) targeting *P*. *vivax* mitochondrial sequences were designed manually. Each set of primers was tested for its ability to amplify *P*. *vivax*-specific DNA. However, only one set of primers (VIV2) targeting the *P*. *vivax* mitochondrial *cox1* gene (Table 2 and Fig 2), was subject to further validation as the other set failed to amplify *P*. *vivax* DNA. The specificity of VIV2 primers was investigated by searching for nucleotide similarity using the BLAST algorithm at the NCBI nucleotide database (www.ncbi.nlm.nih.gov/Blast.cgi) and found to have limited sequence identity only to other *Plasmodium* species. Given the conserved nature of the mitochondrial genome and the sequence similarity across different *P*.*vivax* strains from around the world, no evaluation of VIV2 primers on different *P*.*vivax* strains was performed.

**Table 2:** *P*. *vivax* VIV2 HtLAMP primer sequences (5’ → 3’).

Primer	Sequence
F3	**GGTACTGGATGGACTTTATAT**
B3	**GGTAATGTTAATAATAGCATTACAG**
LF	**GATAACATCTACTGCAACAGG**
LB	**CTACTGTAATGCATCTAAGATC**
FIP	**CCAGATACTAAAAGACCAACCCACCATTAAGTACATCACT**
BIP	**GCTAGTATTATGTCTTCTTTCACTTAATATACCAAGTGTTAAACC**

### Species-specificity of VIV2 LAMP primers

The VIV2 primer set was tested in duplicate on DNA extracts from one PCR-confirmed sample of each of the following species: *P*. *vivax*, *P*. *falciparum*, *P*. *knowlesi*, *P*. *malariae*, *P*. *ovale wallikeri and P*. *ovale curtisi*. There was amplification of *P*. *vivax* and *P*. *knowlesi* DNA but no amplification product was detected for *P*. *falciparum*, *P*. *malariae*, *P*. *ovale wallikeri* or *P*. *ovale curtisi*.

### Quantification of mitochondrial *cox1* gene copy numbers in the *P*. *vivax* genome

**Fig 3:**
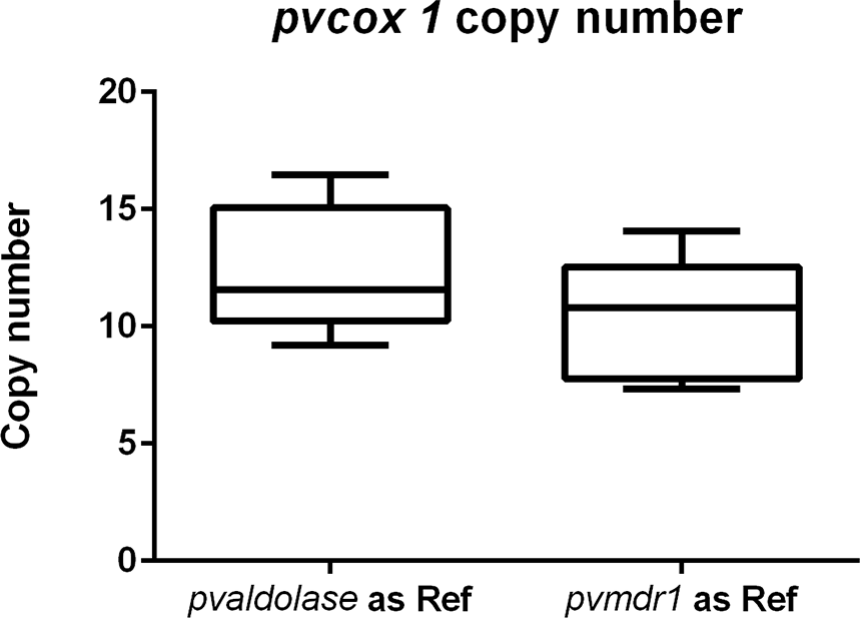
Estimated *Plasmodium vivax* cox1 copy number based on comparison with two single copy genes, *pv aldolase1* and *pvmdr1*.

Using the single-copy *P*. *vivax aldolase* gene as reference, the estimated copy number of *pvcox1* in five selected *P*. *vivax* samples ranged from 9.2–16.47 with a mean of 12.43 (± 1.233). Using the *pvmdr1* gene as reference, the estimated copy number of *pvcox1* ranged from 7.32–14.05 with a mean of 10.28 (± 1.182) (Fig 3).

### Limit of detection of HtLAMP-Pv

Using a two-fold DNA dilution series of a clinical *P*. *vivax* sample, with a starting parasitemia of 90,000 parasites/ μL as determined by quantitative PCR, the LOD was 1.4 parasites/ μL (Table 3). Using the same dilutions, the LOD of *P*. *vivax* LAMP primers published by Han [23] and Patel [30], in the HtLAMP platform was determined to be706 parasites/ μL and 176 parasites/ μL respectively. HtLAMP-Pv performed on the microscopy-determined *P*. *vivax* dilution series in whole blood had an LOD of 2 parasites/ μL (Table 4).

**Table 3:** Analytical sensitivity of HtLAMP-Pv, compared with other published *P*. *vivax* LAMP primers, using a DNA dilution series of clinical sample with qPCR confirmed parasitemia. Each sample was tested in duplicate in the HtLAMP platform using each of the three *P*. *vivax* LAMP primer sets. The dilution at which both duplicates were positive was the limit of detection for each of the primer sets in the HtLAMP platform (Pos* indicates dilutions at which only one of the duplicates was positive). HtLAMP-Pv was able to detect 1.4 parasites/ μL compared with previously published *P*. *vivax* LAMP primers.

Sample	Quantitative PCR (parasites/μL calculated)	[23] 18s rRNA target	[30] Pvr64 target	HtLAMP-Pv *cox1* target
**1 Original**	90,000	Pos	Pos	Pos
**2**	45,000	Pos	Pos	Pos
**3**	22,500	Pos	Pos	Pos
**4**	11,250	Pos	Pos	Pos
**5**	5625	Pos	Pos	Pos
**6**	2813	Pos	Pos	Pos
**7**	1406	Pos	Pos	Pos
**8**	703	Pos	Pos	Pos
**9**	352	Pos*	Pos	Pos
**10**	176		Pos	Pos
**11**	88			Pos
**12**	44			Pos
**13**	22			Pos
**14**	11			Pos
**15**	5.5			Pos
**16**	2.7		Pos*	Pos
**17**	1.4			Pos
**18**	0.7			Pos*
**19**	0.3			

**Table 4:** Analytical sensitivity of HtLAMP-Pv on microscopy-determined whole blood *P*. *vivax* dilution series. The limit of detection HtLAMP-Pv is 2 parasites/ μL, performed in duplicate and where both duplicates were positive.

*P*. *vivax* Parasites /μL	HtLAMP-Pv
**2000**	Pos
**200**	Pos
**20**	Pos
**2**	Pos
**0.2**	Neg
**0**	Neg

### HtLAMP-Pv vs RDT

Quantitative PCR analysis of the *P*. *vivax* blood stage clinical trial sample confirmed a peak parasitemia of 12 parasites/ μL prior to commencement of antimalarial therapy. HtLAMP-Pv was positive and the RDT was negative at this level of parasitemia. The limit of detection (LOD) of the HtLAMP-Pv assay varied depending on the type and volume of sample from which DNA was extracted. The LOD for filter paper extracted using saponin and chelex was more than 12 parasites/ μL whereas the LOD for 10 μL and 50 μL of whole blood extracted using saponin and chelex was 3 parasites/ μL and 1.5 parasites/ μL respectively (Table 5).

**Table 5:** Comparing microscopy and RDT with the limit of detection of HtLAMP-Pv using filter paper and whole blood at 10 μL and 50 μL volumes.

	0.75 parasites/ μL	1.5 parasites/ μL	3.0 parasites/ μL	6.0 parasites/ μL	12 parasites/ μL
**Microscopy**	Neg	Neg	Pos	Pos	Pos
**RDT**	Neg	Neg	Neg	Neg	Neg
**Filter paper (5 μL)**	Neg	Neg	Neg	Neg	Pos
**Whole blood 10 μL**	Neg	Neg	Pos	Pos	Pos
**Whole blood 50 μL**	Neg	Pos	Pos	Pos	Pos

The limit of detection (LOD) of the HtLAMP-Pv assay depends on the starting sample material and the volume of blood extracted. LOD threshold was determined by the presence of 2 positive duplicate tests (ie: 4 positive results) at a particular parasitemia. The LOD for a 5 μL filter paper blood spot extracted using a chelex-saponin protocol was more than 12 parasites/ μL. The LOD for 10 μL of whole blood extracted using a chelex-saponin protocol was 3 parasites/ μL compared with 50 μL of whole blood which was 1.5 parasites/ μL.

### Sensitivity and specificity of HtLAMP-Pv in symptomatic patients

Of the 149 patients with microscopy-confirmed malaria from the district of Kota Marudu, Sabah, Malaysia, 145 were confirmed by PCR: 4 samples were excluded due to lack of microscopy and PCR data, 56 were identified as *P*. *knowlesi* (median parasitemia 2005 parasites/ μL, range 26–143,790), 64 as *P*. *vivax* (median parasitemia 4676 parasites/ μL, range 53–89,640), 7 as *P*. *malariae*, 17 as *P*. *falciparum* (median parasitemia 18,725 parasites/ μL, range 837–693,922) and 1 as a mixed *P knowlesi/P*. *vivax* infection. HtLAMP-Pv was compared with multiplex PCR in these clinical samples. The sensitivity of HtLAMP-Pv for *P*. *vivax* was 95% (62/65, 95% CI 87–99) and specificity was 55% (44/80, 95% CI 43–66) respectively compared with multiplex PCR and 94% (59/62, 95% CI 85–98) and 53% (44/83, 95% CI 42–64) respectively compared with expert microscopy (Table 2). The low specificity of the assay can be attributed to cross-reactivity of the VIV2 primers with *P*. *knowlesi*, with 97% sequence homology at the *cox1* gene between *P*. *vivax* and *P*. *knowlesi*. When *P*. *knowlesi* samples were excluded from the analysis, the specificity was 100% compared with both multiplex PCR and microscopy (Table 6).

**Table 6:** Sensitivity and specificity of HtLAMP-Pv for *P*. *vivax* in symptomatic patients in Sabah with *P*. *falciparum*, *P*. *vivax* and *P*. *knowlesi*. 149 filter paper samples were tested by HtLAMP-Pv; 4 samples were excluded due to a lack of PCR and microscopy data. Among the 145 samples, PCR confirmation of species was as follows: n = 64 *P*. *vivax* n = 56 *P*. *knowlesi*, n = 17 *P*. *falciparum*, n = 7 *P*. *malariae* and n = 1 mixed *P*. *vivax/P*. *knowlesi* infection.

	Sensitivity	Specificity
**HtLAMP-Pv compared with multiplex PCR**	**62/65(95%)**	**44/80 (55%)**
	(95% CI 87–99)	(95% CI 43–66)
**HtLAMP-Pv compared with multiplex PCR excluding Pk samples**	**62/65(95%)**	**24/24 (100%)** (
	(95% CI 87–99)	95% CI 87–99)
**HtLAMP-Pv compared with microscopy**	**59/62 (95%)**	**44/83 (53%)**
	(95% CI 85–98)	(95% CI 42–64)
**HtLAMP-Pv compared with microscopy excluding Pk samples**	**59/62 (95%)**	**18/18 (100%)**
	(95% CI 85–98)	(95% CI 81–100)
**Multiplex PCR compared with microscopy**	**60/65 (92%)**	**78/80 (98%)**
	(95% CI 83–97)	(95% CI 91–99)

### Sensitivity and specificity of HtLAMP-Pv in asymptomatic, reactive active case detection community control patients

HtLAMP-Pv was compared with nested PCR for red cell pellet samples from asymptomatic, microscopy negative community controls from the malaria endemic district of Kota Marudu, Sabah, Malaysia. The sensitivity of HtLAMP-Pv was 71% (95% CI 29–96; 5/7) and specificity was 93% (95% CI 87–97; 98/105) compared with PCR.

### Turnaround time of assay

The HtLAMP assay turnaround time was 1 hour after DNA extraction, 4 hours when combined with whole blood chelex saponin protocol and 6 hours when combined with filter paper rapid DNA extraction protocol.

### Applicability of the assay in a non-reference laboratory in a resource limited setting

HtLAMP-Pv testing of a total of 149 filter paper samples was performed successfully in a regional hospital laboratory in Kota Marudu district, Sabah, Malaysia. Good workflow set up ensured that there was no contamination despite the lack of formal molecular diagnostic infrastructure. Locally trained staff was able to perform and interpret results of the HtLAMP-Pv assay using only a centrifuge, pipettes, water bath and a portable spectrophotometer.

## Discussion

Field-applicable diagnostic tools for the detection of *Plasmodium vivax* are essential components for the malaria eradication agenda [44]. Given the widespread distribution and unique challenges *P*. *vivax* poses compared with *P*. *falciparum*, there is a pressing need for the development of species- specific molecular diagnostic tools. LAMP is a molecular diagnostic tool which holds much promise in terms of its ability to be deployed in non-referral laboratory settings, given its simplicity and rapid assay turnaround time, ability to be performed on crudely extracted DNA from both whole blood and filter paper and lack of expensive equipment. The colourimetric, 96 well microtitre plate-based platform for performing LAMP (HtLAMP) for the detection of *Plasmodium* parasites, as previously described [43], increases the throughput of the LAMP using minimal equipment. The objective of this paper was to develop and validate a *P*. *vivax* specific HtLAMP assay on this platform with good diagnostic accuracy.

The 6-kb mitochondrial genome of the genus *Plasmodium* encodes three mitochondrial proteins- cytochrome B (*cytb*) and subunit 1 and 3 of cytochrome c oxidase (*cox1* and *cox3*), and is estimated to be present in relatively high copy number. The complete mitochondrial genome of *P*. *vivax* (Genbank AY598035) has been shown to be closely related to *P*. *knowlesi* [45]. Previously published standard PCR primers for *P*. *vivax* targeting *cox1* have shown 100% sensitivity and specificity [46], but were not evaluated against *P*. *knowlesi*. LAMP primers targeting mitochondrial sequences for the detection of *P*. *genus* and *P*. *falciparum* demonstrated an analytical sensitivity of 5 parasites/ μL [27] suggesting that mitochondrial DNA offers an attractive target, presumably due to increased copy number of mitochondrial targets within cells. Recent estimates of genomic sequence coverage have shown that the *P*. *falciparum* genome contains ~20 copies/cell of the mitochondrial genome [47].

This HtLAMP-Pv assay targeting the conserved *cox1* gene demonstrated excellent analytic sensitivity, being able to detect 1.4 parasites/ μL. This is the lowest LOD so far achieved for a published *P*. *vivax–*specific LAMP assay. The estimated copy number for *cox1* in *P*. *vivax* is approximately 11 copies/ cell. Therefore, it is likely that the sensitivity of the HtLAMP-Pv assay is a reflection of the increased number of mitochondrial targets per cell. Previously published *P*. *vivax* LAMP primers, which targeted non-mitochondrial genes, when used in the HtLAMP platform had limits of detection of 706 parasites/ μL and 176 parasites/ μL which correlated well with published limits of detection of 125–500 parasites/ μL for these *P*. *vivax* primers sets [30].

The *pkcox1* gene of *P*. *knowlesi* exhibits 97% sequence identity with *pvcox1* at the nucleotide level, and thus the cross-reactivity of the VIV2 primers between these two species was expected. However, there was no cross-reactivity with *P*. *falciparum* (87% sequence identity), *P*. *ovale wallikeri* (92%), *P*. *ovale curtisi* (92%) or *P*. *malariae* (93%).

Validation of the HtLAMP-Pv in clinical samples of symptomatic patients with vivax, falciparum and knowlesi malaria demonstrated sensitivity for *P*. *vivax* of 94–95% and a specificity of 53–55% compared with microscopy and multiplex PCR respectively. The poor specificity however was a reflection of the cross-reactivity with *P*. *knowlesi*. When *P*. *knowlesi* samples were excluded from the analysis, the specificity of the HtLAMP-Pv assay improved to 100%, compared with both multiplex PCR and microscopy.

While this cross-reactivity appears to be a limitation of this HtLAMP-Pv assay, *P*. *knowlesi* malaria is uncommon or absent in most areas of *P*. *vivax* endemicity, so this would be an important consideration only in Malaysia, where *P*. *knowlesi* predominates [48] and in the other countries in south-east Asia where *P*. *knowlesi* human infection has been documented [49]. In terms of treatment, both *P*. *vivax* and *P*. *knowlesi* respond to artemisinin-based combination therapy (ACT) [50]. However, in elimination programmes utilising primaquine for radical cure of *P*. *vivax* malaria, there is a potential risk of inappropriate use of this potentially haemolytic drug in people with *P*. *knowlesi* infections. This is a problem localised to Southeast Asia, and would not pose a problem for LAMP-based detection and radical treatment of *P*. *vivax* for malaria elimination elsewhere.

The HtLAMP-Pv assay was also evaluated in a limited sample set of asymptomatic, microscopy negative, community control patients enrolled from the same village as a case patient, as a result of reactive active case detection. Although the LOD of HtLAMP-Pv appears to be 1.4 parasites/ μL, in this sample set its sensitivity was only 71%. This may be due to the very low parasitemias in these 7 PCR positive samples or variability due to stochastic effects at such low parasitemias. Therefore further validation in a larger sample set is required to confirm the sensitivity of HtLAMP-Pv in this population in order to evaluate the potential role for HtLAMP-Pv as a diagnostic tool in malaria elimination settings.

HtLAMP-Pv showed significantly better sensitivity than the SD Bioline Pf/Pan RDT at low parasitemia. RDTs were negative in the serially diluted samples at 12 parasites/ μL. HtLAMP-Pv was positive at this level. The analytical sensitivity of the assay varied depending on whether filter paper samples or whole blood was used irrespective of the chelex-saponin DNA extraction protocol used. This may have important implications for choosing the type of sample collected in addition to choosing the appropriate diagnostic tool for surveillance or screening policy and protocols for malaria elimination programs.

In this study we also demonstrated that HtLAMP-Pv performed well in the 96-well microtitre plate platform for increasing the throughput of the assay in a non-referral laboratory in a district hospital in Sabah, Malaysia. DNA extraction was performed in the non-referral laboratory using a chelex protocol on filter paper blood spots and the HtLAMP-Pv assay was able to process these samples in a simple water-bath within one hour from time of DNA extraction. Positive and negative results were readily identified by two locally trained staff by visual inspection of the colour change. Optical densitometry readings at 600 nm in portable photospectrometer were used to provide objective confirmation of the visually detected results. As such the validation of this HtLAMP-Pv assay in a rural district laboratory setting confirms the potential it has as a field-applicable molecular diagnostic tool. Furthermore, the process of assay validation using the combination of visual and optical densitometry values has previously shown that the visually detectable colour change was reliable for determining both positive and negative results [43]. Therefore while the photospectrometer offers objective confirmation, it is not an essential component of the assay.

Some of the limitations to this platform pertain to DNA extraction. Firstly, in order to maintain cost effectiveness of the assay, modified chelex-based protocols were used for whole blood and filter paper extractions. Although these multi-step DNA extraction processes, which relied on a centrifuge, were performed adequately in a resource limited setting, further simplification of DNA extraction would enhance the feasibility of the HtLAMP-Pv assay. It would also allow a greater number of samples to be processed, as might be required for mass surveillance for malaria elimination, thereby making full use of the high throughput aspect of the HtLAMP platform. Secondly, while equivalent small volumes of blood on filter paper and whole blood have shown whole blood to produce better analytical sensitivity in the HtLAMP platform [43], the limit of detection of HtLAMP-Pv using larger volumes of blood on filter paper is yet to be established.

In conclusion, this study outlines the development and validation of a novel *P*. *vivax*-specific LAMP assay which combines a low limit of detection with a high throughput, colourimetric, field applicable molecular diagnostic assay. As such, this HtLAMP assay holds much promise as a diagnostic tool to support malaria elimination efforts in resource-limited *P*. *vivax* endemic settings.
